# Milk Casein Inhibits Effect of Black Tea Galloylated Theaflavins to Inactivate SARS-CoV-2 In Vitro

**DOI:** 10.3390/bioengineering10091068

**Published:** 2023-09-09

**Authors:** Maiko Nakashio, Eriko Ohgitani, Masaharu Shin-Ya, Masaya Kawamoto, Masaki Ichitani, Makoto Kobayashi, Takanobu Takihara, Hitoshi Kinugasa, Hiroyasu Ishikura, Osam Mazda

**Affiliations:** 1 Department of Immunology, Kyoto Prefectural University of Medicine, Kyoto 602-8566, Japan; smapmaiko0428@gmail.com (M.N.);; 2 Department of Emergency and Critical Care Medicine, Faculty of Medicine, Fukuoka University, Fukuoka 814-0180, Japan; 3 Department of Molecular Anti-Virus Immunology, Kyoto Prefectural University of Medicine, Kyoto 602-8566, Japan; 4 Central Research Institute, ITO EN, Ltd., Shizuoka 421-0516, Japan

**Keywords:** COVID-19, Omicron mutant, black tea, Flavan-3-ols, theaflavin digallate, theaflavin monogallate, polyphenol, casein micelle, saliva

## Abstract

Continuing caution is required against the potential emergence of SARS-CoV-2 novel mutants that could pose the next global health and socioeconomical threats. If virus in saliva can be inactivated by a beverage, such a beverage may be useful because the saliva of infected persons is the major origin of droplets and aerosols that mediate human-to-human viral transmission. We previously reported that SARS-CoV-2 was significantly inactivated by treatment in vitro with tea including green tea and black tea. Catechins and its derived compounds galloylated theaflavins (gTFs) bound to the receptor-binding domain (RBD) of the S-protein and blocked interaction between RBD and ACE2. Black tea is often consumed with sugar, milk, lemon juice, etc., and it remains unclarified whether these ingredients may influence the anti-SARS-CoV-2 effect of black tea. Here, we examined the effect of black tea on Omicron subvariants in the presence of these ingredients. The infectivity of Omicron subvariants was decreased to 1/100 or lower after treatment with black tea for 10 s. One or two teaspoons of milk (4~8 mL) completely blocked the anti-viral effect of a cup of tea (125 mL), whereas an addition of sugar or lemon juice failed to do so. The suppressive effect was dose-dependently exerted by milk casein but not whey proteins. gTFs were coprecipitated with casein after acidification of milk-supplemented black tea, strongly suggesting the binding of gTFs to casein. The present study demonstrates for the first time that an addition of milk cancelled the anti-SARS-CoV-2 effect of black tea due to binding of casein to gTFs.

## 1. Introduction

Oral tissues including salivary glands and oral mucosa are important regions of SARS-CoV-2 infection in humans [1,2]. SARS-CoV-2 is mainly transmitted by droplets and aerosols that are derived from the saliva of COVID-19 patients and asymptomatic infected persons, although bronchial and laryngeal aerosols may also be involved in the viral transmission [3,4,5,6,7,8,9,10]. Saliva containing the virus is scattered by speaking, coughing and sneezing from the oral cavity to environment and forms the droplets and aerosols that could reach the nasal and oral mucosa of nearby persons to cause infection. We considered that the inactivation of virus in saliva may result in the attenuation of viral spread among people, and explored various food ingredients that inactivate SARS-CoV-2. We reported that tea, including green tea, roasted green tea, oolong tea and black tea, significantly reduced the infectivity of conventional SARS-CoV-2 in vitro [11,12]. We also found that (-) epigallocatechin-gallate (EGCG) rapidly and powerfully inactivated the virus. EGCG is a tea catechin compound contained at high concentrations in tea leaves (10–15% dry weight) and green tea (44–84 mg per 100 mL) [13]. Similar effects were also exerted by black tea ingredients, theaflavins (TFs) with (a) 3 and/or 3′ galloyl moiety(ies) (theaflavin-3-gallate (TF3G), theaflavin-3′-gallate (TF3′G), theaflavin-3,3′-O-digallate (TFDG)) and theasinensin A (TSA), which are produced from catechins by enzymatic oxidation during the manufacturing of black tea [11]. As for the mechanisms underlying the viral inactivation effect, we showed that EGCG, TSA and TFDG bound to the receptor-binding domain (RBD) of viral S-protein [11]. The binding inhibited the interaction between RBD and the human receptor angiotensin converting enzyme 2 (ACE2). Therefore, the virus treated with EGCG, TSA or TFDG could not infect cells through the RBD and ACE2 interaction. Tea inactivated SARS-CoV-2 even in the presence of human saliva [12].

More than 70% of tea is manufactured into black tea that is consumed all over the world. People in some countries/areas consume larger amounts of black tea per capita than those in other countries/areas, but any epidemiological analysis has not demonstrated a significant negative correlation between black tea consumption and the rate or severity of infection. Black tea is often consumed with sugar, milk, lemon juice, honey, jam, cinnamon, and so on. It remains to be elucidated whether these ingredients could potentially influence the activity of black tea to block the virus infection.

In this study, we aimed at clarifying whether an addition of sugar, milk, or lemon juice influenced the anti-viral effect of black tea. As results, we found that milk, but not sugar nor lemon juice, inhibited anti-viral activity of black tea. Thus, we investigated the inhibitory molecule in milk in more detail. The virus was diluted in human saliva before the treatment with tea, to simulate what may happen in the oral cavity of a SARS-CoV-2-infected person who ingests tea.

## 2. Materials and Methods

### 2.1. Virus, Cells, and Culture Medium

The SARS-CoV-2 strains shown in Supplementary Table S1 were kindly provided from the Japan National Institute of Infectious Diseases (Tokyo, Japan) and propagated using VeroE6/transmembrane serine protease 2 (VeroE6/TMPRSS2) cells [14] that were obtained from the Japanese Collection of Research Bioresources Cell Bank, National Institute of Biomedical Innovation (Osaka, Japan). Cells were cultured in Dulbecco’s Modified Eagle’s Medium (DMEM) supplemented with geneticin (G418) disulfate (1 mg/mL), penicillin (100 units/mL), streptomycin (100 μg/mL) and 5% fetal bovine serum at 37 °C in a 5% CO_2_/95% humidified atmosphere (standard conditions).

### 2.2. Reagents

Black tea infusions were prepared by soaking 40 g of ground and homogenized tea leaves in 2000 mL water at 80 °C for 30 min (original black tea). The relatively long infusion time was chosen to avoid the nonspecific inhibitory effect of milk, etc. Concentrations of TFs in the tea infusion are shown in the Supplementary Table S2. After centrifugation at 4000 rpm for 15 min, supernatants were collected and filtrated through Toyo No. 2 filter papers, followed by evaporation and freeze-drying. TF, TF3G, TF3′G and TFDG were purchased from FUJIFILM Wako Pure Chemical Corporation (Osaka, Japan). Milk, sugar and lemon were purchased at a grocery store in Kyoto. Hammarsten bovine casein was purchased from Sigma-Aldrich (St. Louis, MO, USA). Whey protein solution was prepared as described with slight modifications [15,16]. Briefly, milk was heated to 70 °C followed by an addition of citric acid at 0.2% (*w*/*v*) and immediate agitation. After incubation for 10 min, precipitates were removed by filtration, and supernatant was used as a solution of whey proteins. Bovine albumin, human albumin and ovalbumin were purchased from FUJIFILM Wako Pure Chemical Corporation. Human saliva was purchased from Lee Biosolutions (Maryland Heights, MO, USA) (Supplementary Table S3) [12].

### 2.3. Virus Treatment and TCID_50_ Assay

Freeze-dried powders of black tea extract were dissolved in sterilized distilled water at 78 °C to prepare an ×1 concentration of original tea. After chilling at room temperature, each solution was supplemented with sugar (6 g per 160 mL of tea), milk (40 mL per 160 mL of tea) and/or lemon juice (2 mL per 160 mL of tea). In some experiments, each tea solution was supplemented with various volumes of 2.7% (*w*/*v*) solution of casein, 0.7% (*w*/*v*) solution of whey protein, 5% (*w*/*v*) ovalbumin, 5% (*w*/*v*) bovine albumin or 5% (*w*/*v*) human albumin. Each sample, including each tea and protein solution, was passed through a 0.45 μm filter. Human saliva was sterilized by UV irradiation for 30 min. Virus suspension (3.0 × 10^5^ TCID_50_/5 μL) was mixed with 45 μL of saliva, followed by an addition of tea at 1:1 (vol:vol) for 10 s. Immediately, the virus/saliva/tea mixture was serially diluted 10-fold with MS in 96-well plates. Each sample was chilled on ice, and 50 μL of the sample was added to the VeroE6/TMPRSS2 cells in each well (in advance, the VeroE6/TMPRSS2 cells had been seeded into 96-well plates at 5 × 10^4^/100 μL/well (N = 4) and cultured for 24 h). After culture for 3 days, the cytopathic effect (CPE) (cell death caused by Omicron variants of SARS-CoV-2) was observed under a phase contrast microscope [17].

### 2.4. Calculation of TCID_50_ Values

TCID_50_ values were calculated by the Reed–Muench method as described elsewhere [11]. If one or more triplicate wells of the lowest dilution of a sample did not show CPE, the highest possible average of TCID_50_ value was calculated for the sample.

### 2.5. Measurement of Concentrations of TFs

Black tea was supplemented with 25% (*v*/*v*) bovine milk followed by an addition of citric acid to a final concentration of 0.2% (*w*/*v*). Precipitates were removed by filtration and centrifugation. Concentrations of TF, TF3G, TF3′G and TFDG in the supernatant were measured by HPLC/MS using an LCMS-8040 and LC-2040 (Shimazu, Kyoto, Japan) under the following conditions. Column: InertSustain C18 2 µm, 2.1 I.D. × 50 mm; mobile phase: acetonitrile/ethyl acetate/water/formic acid = 21/3/75/1 (*v*/*v*/*v*/*v*), isocratic elution; flow rate: 0.2 mL/min; column temp: 40 °C; detector: MS. Un-supplemented black tea was also tested.

### 2.6. Statistical Analysis

Statistical significance was analyzed by Tukey’s multiple comparison test and ANOVA (analysis of variance) using GraphPad Prism 9 manufactured by GraphPad Software (San Diego, CA, USA). *p* < 0.05 was considered significant. All experiments were repeated twice, and reproducible data were obtained.

## 3. Results

### 3.1. Milk Prevented Inactivation of SARS-CoV-2 by Black Tea

**Figure 1:**
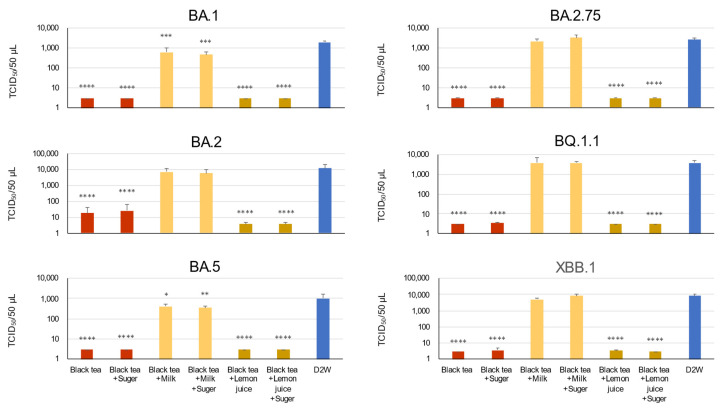
An addition of milk cancelled the inactivation effect of black tea on Omicron variants of SARS-CoV-2. Black tea was supplemented with milk, sugar, and/or lemon juice as described in the Materials and Methods. Indicated virus was diluted in saliva from healthy individuals. The tea was added to the virus/saliva for 10 s, immediately followed by a serial dilution with MS. TCID_50_ assay was performed as described in the Materials and Methods. Virus titer of each sample (means ± S.D.) is shown (*n* = 3). * *p* < 0.05, ** *p* < 0.01, *** *p* < 0.001 and **** *p* < 0.0001 vs. Control (DW) by Tukey’s multiple comparison test and ANOVA.

Omicron subvariant strains of SARS-CoV-2 (Supplementary Table S1) were diluted in human saliva (Supplementary Table S3) and treated with black tea for 10 s in vitro. Consistent with our previous studies [11,18], the viruses were significantly inactivated by black tea, as shown in Figure 1. We supplemented black tea with sugar, milk and/or lemon juice at ratios comparable to ordinary beverages (6 g sugar, 40 mL milk and 2 mL lemon juice per 160 mL of tea), and examined whether the supplementation influenced the ability of black tea to inactivate the viruses diluted in human saliva. Although black tea significantly inactivated the SARS-CoV-2 variants, the anti-viral activity of the black tea was totally hampered by an addition of milk (Figure 1). Neither sugar nor lemon juice significantly affected the titers of the virus.

### 3.2. Casein Prevented Inactivation of SARS-CoV-2 by Black Tea

**Figure 2:**
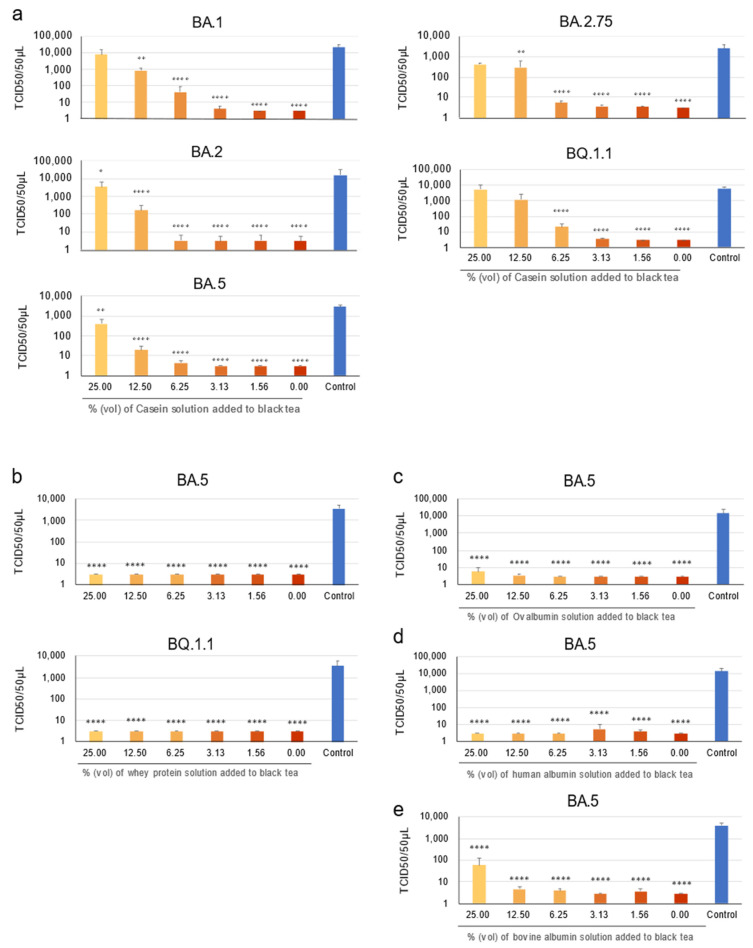
Milk casein abrogated anti-SARS-CoV-2 effect of black tea. (**a**) Casein solution at a concentration of 2.7% (*w*/*v*) (comparable to a typical concentration of casein in ordinary cow’s milk) was added to black tea at the indicated % (vol) (vol of black tea was regarded as 100%). The mixtures were added to the virus/saliva for 10 s, and TCID_50_ assay was performed as described in the Materials and Methods. Virus titer of each sample (means ± S.D.) is shown (n = 3). (**b**–**e**) Whey protein solution (cow’s milk deprived of casein) (**b**) and 5% (*v*/*v*) solutions of ovalbumin (**c**), human albumin (**d**) and bovine albumin (**e**) were added to black tea at the indicated % (vol) (vol of black tea was regarded as 100%). The mixtures were added to the virus/saliva for 10 s, and TCID_50_ assay was performed as above. * *p* < 0.05, ** *p* < 0.01 and **** *p* < 0.0001 vs. Control (DW) by Tukey’s multiple comparison test and ANOVA.

What is the active component in milk that inhibits the anti-SARS-CoV-2 activity of black tea? Milk contains various proteins, among which caseins are contained at the highest concentrations [19]. Some previous literature reported that caseins form a complex with tea catechins [20,21,22,23]. Because gTFs are derived from catechins and share similar molecular structures with them, we hypothesized an involvement of casein in the virus inhibitory activity of milk. We prepared a casein solution at a concentration of 2.7% (*w*/*v*) comparable to a standard concentration of casein in ordinary cow’s milk (Figure 2a). For example, an addition of 25% (vol) of this casein solution to black tea (thus, 250 µL casein solution was added into 1 mL of tea) simulates an addition of 25 mL of milk to 100 mL of black tea, resulting in an approximate casein concentration of 0.54% (*w*/*v*). The results were that casein dose-dependently prevented black tea from reducing the virus titer (Figure 2a).

### 3.3. Anti-Viral Activity of Black Tea Was Not Suppressed by Other Proteins. including Whey Proteins

We examined whether casein specifically suppressed the effect of black tea on SARS-CoV-2 or if the suppressive activity was also exerted by other proteins. Milk proteins deprived of casein (whey proteins) were mixed with the virus in human saliva and virus titers were examined as above. As shown in Figure 2b, both BA.5 and BQ.1.1 viruses were drastically inactivated by the treatment with black tea, even in the presence of whey protein at all the concentrations that we tested (~0.54%).

Similar experiments were also performed using ovalbumin and bovine and human albumins instead of the milk proteins. Ovalbumin and human albumin failed to neutralize the inactivation effect of tea on BA.5 (Figure 2c,d). The anti-viral effect was partially suppressed by bovine albumin at the highest concentration (0.54%) (Figure 2e).

### 3.4. TFs Bind to Casein

Finally, we asked whether gTFs bound to casein, like catechins that have been reported to do so [22,23,24,25]. Milk casein in tea/milk was precipitated by an addition of 0.2% (*w*/*v*) citric acid. Quantification of TFs in the supernatant showed that concentrations of TF and gTFs were reduced by more than 90% and more than 99%, respectively (Supplementary Table S2). Thus, gTFs in tea/milk were coprecipitated with casein, strongly suggesting the binding of gTFs to casein.

## 4. Discussion

In this study we demonstrated that the inactivation effect of black tea on SARS-CoV-2 was counteracted by an addition of milk casein into the black tea in vitro. An addition of sugar or lemon juice did not influence the anti-viral effects of black tea, suggesting the absence of inhibitory molecules in sugar and lemon juice.

Bovine milk contains 32–37 g/L of proteins, and caseins account for about 80% of the whole milk proteins [26,27]. Caseins consist of four polypeptides, i.e., alpha_S1_-, alpha_S2_-, beta- and kappa-caseins, and form large colloidal particles, the casein micelles, that are dispersed in milk [26,27,28]. Caseins are involved in the transport of calcium and phosphorus. Previous reports indicated that kappa-casein bound to human rotavirus and inactivated it [29]. Casein phosphopeptides derived from alphaS1-, alphaS2-, and beta-caseins stimulated host cells to produce interferon and rendered the cells resistant to feline calicivirus [30]. However, it has not been reported that caseins bind to gTFs and hamper their anti-viral activities.

Previous reports demonstrated that caseins bound to catechins including EGCG [22,23,24,25], leading to a significant decrease in the antioxidant activity of catechins and non-galloylated TF [22,25]. Addition of milk also blocked the vascular protection effect of black tea [24]. Our results indicated that gTFs also bind to caseins (Supplementary Table S2) which may have resulted in a lack of the anti-SARS-CoV-2 effect of black tea supplemented with milk (Figure 1 and Figure 2a).

TFs are involved in flavor, bitter taste and astringency of tea [20,21]. Addition of milk reduces the bitterness and astringency of tea, probably due to the binding of caseins to catechins and TFs.

Tea drinkers add various amount of milk to their black tea infusions. In our study, an addition of 3.31% to 6.25% (*v*/*v*) of 2.7% casein solution into black tea (final concentrations of casein were 0.086% to 0.16%) totally blocked the inactivation effect against Omicron subvariants (Figure 2a). In other words, one or two teaspoons of milk (4 to 8 mL) completely blocked the anti-viral effect of a cup of tea (125 mL). A smaller volume of milk partially reduced the anti-viral activities. Needless to say, the relationship between the ratios of milk vs. tea infusions and degrees of counteraction of anti-SARS-CoV-2 effect may differ depending on the types of tea and milk.

The Omicron subvariants of SARS-CoV-2 have some amino acid substitutions in the viral spike proteins [31,32,33,34,35], which may contribute to the high contagiousness of the variants. We previously showed that TFDG may bind to the receptor-binding domain (RBD) of the spike proteins of conventional SARS-CoV-2 [11]. More recently, we found that the virus inhibitory effect of TFDG may be influenced by some specific amino acid substitutions in the Omicron RBDs, including G446S and F490S (manuscript submitted).

The present findings that black tea inactivates SARS-CoV-2 only in the absence of milk may partly explain the reason why the rate and severity of infection in a country/area are not negatively correlated with black tea consumption in the country/area, although various factors including vaccination rates and geographical, genetical, cultural, and social factors influence the degree of virus spread among a particular population.

Ingestion of tea by infected persons at appropriate occasions may potentially attenuate viral spread to nearby persons, as we previously discussed [11,12], and black tea without milk may be applicable to this purpose. Besides, repetitive ingestion of milk-free black tea shortly after SARS-CoV-2 infection could potentially bring a beneficial effect on disease progression in infected persons, because viral amplification in the oral cavity at the earliest stages of infection may play important roles in subsequent viral infection in the lung and other organs [1]. Clinical studies are required to investigate these possibilities.

## 5. Conclusions

Omicron subvariant viruses significantly lost infectivity after treatment with black tea for 10 s. But the anti-viral activity was prevented by supplementation of milk into the black tea in a dose-dependent manner. Supplementation of sugar, lemon juice or whey proteins did not interfere with the anti-viral effect of black tea. Milk casein bound to TFs that otherwise contributed to the virus inactivation effect. These results suggest the possibility that intake of black tea without milk by infected persons may result in inactivation of the virus in saliva.

## Data Availability

The datasets used and/or analyzed during the current study are available from the corresponding author on reasonable request.

## Supplementary Materials

The following supporting information can be downloaded at: https://www.mdpi.com/article/10.3390/bioengineering10091068/s1, Table S1: Viruses used in the study. Table S2: Concentrations of theaflavins in black tea and black tea deprived of casein. Supplementary Table S3: Donors of saliva used in the study.
