# Supplementary material for: Milk Casein Inhibits Effect of Black Tea Galloylated Theaflavins to Inactivate SARS-CoV-2 In Vitro
Source: Bioengineering (Basel). 2023 Sep 9;10(9):1068. doi: 10.3390/bioengineering10091068 (PMC10526027; doi:10.3390/bioengineering10091068)
Supplement: Supplementary file 1 [file bioengineering-10-01068-s001.zip › bioengineering-2592850-supplementary.pdf]

Supplementary Table S1

Pango lineage	Strain	GISAID ID
BA.1.18	hCoV-19/Japan/TY38-873/2021	EPI_ISL_7418017
BA.2	hCoV-19/Japan/TY40-385/2022	EPI_ISL_9595859
XE	hCoV-19/Japan/TY41-686/2022	EPI_ISL_12703378
BA.5	hCoV-19/Japan/TY41-702/2022	EPI_ISL_13241867
BA.2.75	hCoV-19/Japan/TY41-716/2022	EPI_ISL_13969765
XBB.1	hCoV-19/Japan/TY41-795-P1/2022	EPI_ISL_15669344
BQ.1.1	hCoV-19/Japan/TY41-796-P1/2022	EPI_ISL_15579783

Supplementary Table S1
Viruses used in the study. All viruses were kindly provided by Japan National Institute of Infectious Diseases (Tokyo, Japan).

Supplementary Table S2

	TF	TF3G	TF3'G	TFDG
Concentration in black tea	11.3 (μM）	16.7 (μM）	9.8 (μM）	24.3 (μM）
Concentration in black tea deprived of casein	1.1 (μM）	<0.1 (μM）	<0.1 (μM）	<0.1 (μM）
% Reduction due to casein deprivation	90.3%	>99.4%	>99.0%	>99.6%

Supplementary Table S2
Concentrations of theaflavins in black tea and black tea deprived of casein.

Supplementary Table S3

Number	Birth year	Gender	Race
1	1962	Male	Caucasian
2	1986	Female	Caucasian
3	1981	Female	Caucasian
4	1987	Male	Caucasian
5	1989	Male	African American
6	1977	Female	Caucasian
7	1967	Female	Caucasian
8	1991	Male	Caucasian
9	1937	Male	Caucasian
10	1968	Male	Caucasian
11	1970	Female	Caucasian
12	1986	Male	Caucasian
13	1984	Female	Caucasian
14	1992	Male	African American
15	1979	Male	Caucasian
16	1955	Female	African American
17	1980	Female	African American
18	1978	Female	Caucasian
19	1992	Male	Caucasian

Supplementary Table S3 Donors of saliva used in the study. Saliva was purchased from Lee Biosolutions (Maryland Heights, MO, USA).
